# Closing the Gap Between Therapeutic Use and Mode of Action in Remedial Herbs

**DOI:** 10.3389/fphar.2019.01132

**Published:** 2019-10-03

**Authors:** Joaquim Olivés, Jordi Mestres

**Affiliations:** ^1^Research Group on Systems Pharmacology, Research Programme on Biomedical Informatics (GRIB), IMIM Hospital del Mar Medical Research Institute, Barcelona, Spain; ^2^Department of Experimental and Health Sciences, University Pompeu Fabra, Barcelona, Spain

**Keywords:** ethnopharmacology, traditional medicine, network pharmacology, mechanism of action, phytochemicals, endogenous metabolites, plant metabolomics

## Abstract

The ancient tradition of taking parts of a plant or preparing plant extracts for treating certain discomforts and maladies has long been lacking a scientific rationale to support its preparation and still widespread use in several parts of the world. In an attempt to address this challenge, we collected and integrated data connecting metabolites, plants, diseases, and proteins. A mechanistic hypothesis is generated when a metabolite is known to be present in a given plant, that plant is known to be used to treat a certain disease, that disease is known to be linked to the function of a given protein, and that protein is finally known or predicted to interact with the original metabolite. The construction of plant–protein networks from mutually connected metabolites and diseases facilitated the identification of plausible mechanisms of action for plants being used to treat analgesia, hypercholesterolemia, diarrhea, catarrh, and cough. Additional concrete examples using both experimentally known and computationally predicted, and subsequently experimentally confirmed, metabolite–protein interactions to close the connection circle between metabolites, plants, diseases, and proteins offered further proof of concept for the validity and scope of the approach to generate mode of action hypotheses for some of the therapeutic uses of remedial herbs.

## Introduction

Plant leaves, roots, barks, and extracts have been used since the dawn of human history to treat various discomforts and maladies. The healing properties of remedial herbs were most likely identified through a long and serendipitous learning process that once acquired was carefully passed through generations. Still today, traditional medicines represent a well-established therapeutic alternative to synthetic drugs in vast parts of the world (Tao et al., 2014). However, there is still a profound lack of understanding about the specific chemical ingredient(s) and the exact mechanism(s) of action by which medicinal plants exert their therapeutic effect.

**Figure 1:**
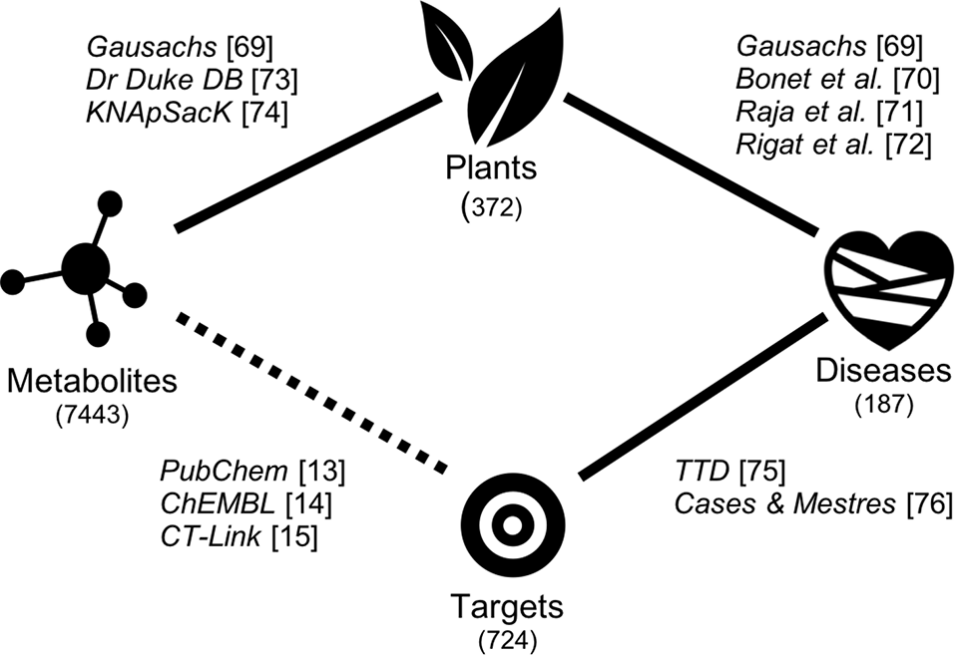
Scheme showing the data sources and the process of closing the gap between the therapeutic use of plants and the protein targets predicted for the endogenous plant metabolites.

In recent years, global efforts to generate, collect, store, and make publicly available data connecting plants with their endogenous metabolites (phytoconstituents), interacting proteins, and disease indications have set the ground to develop novel systems approaches to unveiling the mode of action of remedial herbs (Liu et al., 2013; Lagunin et al., 2014; Chen et al., 2017). This is schematically illustrated in **Figure 1**. A number of publicly available well annotated databases on medicinal plants in use in different regions of the planet exist already (Chen, 2011; James et al., 2013; Ntie-Kang et al., 2013; Tota et al., 2013; Pathania et al., 2015; Mohanraj et al., 2018). Once data connecting the different aspects of ethnopharmacological relevance are known, the circle is closed and mechanistic hypotheses emerge naturally. The problem arises when gaps of data exist and the circles cannot be closed. In this respect, most current ethnomedicinal studies still focus on which parts of the plant are used to treat common ailments (Chassagne et al., 2016). Initiatives to identify and isolate some of the chemical structures present in those parts of therapeutic interest are expensive and inefficient. This notwithstanding, at least 50,000 endogenous plant metabolites have been already identified (Hounsome et al., 2008).

However, *in vitro* affinity data between plant metabolites and human proteins are scarce to find in public repositories (Bolton et al., 2008; Gaulton et al., 2012). Therefore, more efforts are needed in this direction to close the gap between therapeutic use and mode of action in remedial herbs (represented as a dotted line in **Figure 1**). One option is to process large libraries of isolated small molecules from plants through *in vitro* high-throughput screening assays to identify affinities for therapeutically relevant proteins. This is a highly tedious and expensive endeavor if one wants to be comprehensive. Alternatively, modern state-of-the-art computational methods to predict the affinity of small molecules across thousands of proteins can be used to prioritize any further *in vitro* testing of selected small molecules on particular proteins (Vidal et al., 2011; Garcia-Serna et al., 2015). Applications on predicting the targets of natural medicines are increasingly being reported (Keum et al., 2016; Fang et al., 2017; Sawada et al., 2018; Yi et al., 2018).

The last step involves connecting those confirmed interacting proteins with the actual disease for which the plant is prescribed. This task is now facilitated by the recent construction of databases connecting human genes with diseases (Piñero et al., 2017). The aim of this work is to collect and integrate all pieces of data and processes that allow for automatically generating mechanistic hypotheses for the known therapeutic uses of plants.

## Results and Discussion

Among the 372 medicinal plants present in our integrated database, *Sambucus nigra* (black elder) is the plant associated with the highest number of therapeutic uses (31). It is recommended for bronchitis, migraine, diarrhea, nausea, hyperuricemia, and influenza, to name just a few. Genus *Sambucus* belongs to the Caprifoliaceae family of flowering plants, whose leaves, flowers, and berries are traditionally used worldwide for a wide variety of medicinal applications (Dulf et al., 2013; Mahmoudi et al., 2014). Following *Sambucus nigra* in the list of plants with widest therapeutic use are *Allium sativum* (24), *Rosmarinus officinalis* (22), *Mentha spicata* (21), *Urtica dioica* (21), *Salvia officinalis* (21), and *Thymus vulgaris* (21), all of them found easily in many parts of the world and used as food and/or spice.

**Figure 2:**
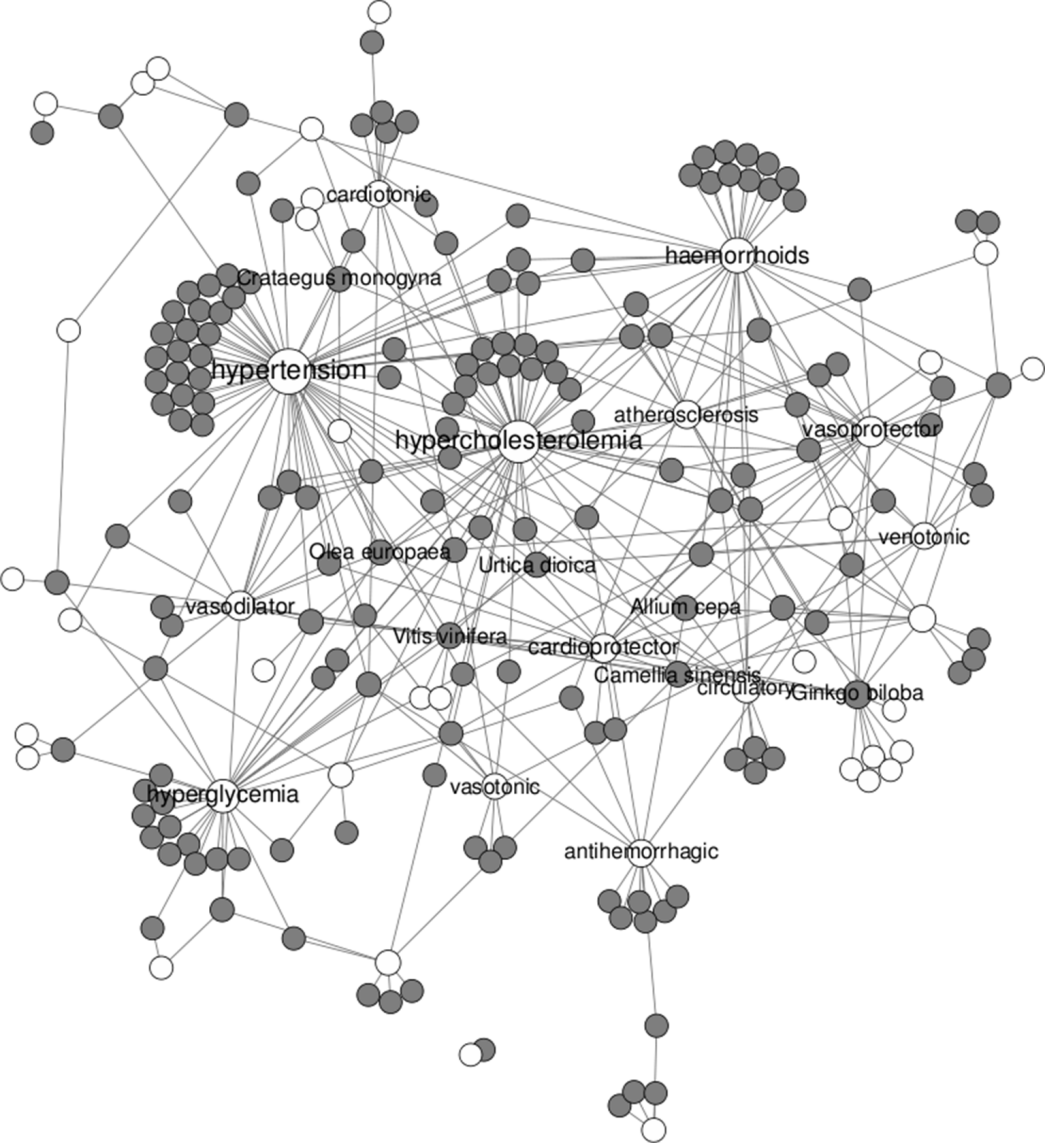
Network of remedial herbs (gray circles) linked to therapeutic uses in cardiovascular diseases (white circles).

If we focus on cardiovascular diseases, a total of 171 plants were found to be associated with 46 different therapeutic uses. For illustrative purposes, the network of plants linked to cardiovascular diseases is shown in **Figure 2**. Among those, *Ginkgo biloba* is the plant with the most cardiovascular uses (with 14), followed by *Camellia sinensis* (with 8) and *Allium cepa*, *Crataegus monogyna*, *Olea europea*, *Urtica doica*, and *Vitis vinifera* (with 7). Among diseases, hypertension, hypercholesterolemia, hyperglycemia, and haemorrhoids are clearly the cardiovascular aspects being most addressed by remedial herbs.

*Ginkgo biloba* and *Camellia sinensis* are indigenous plants from Asia (Cybulska-Heinrich et al., 2012; Moore et al., 2009). The extracts of the leaves and nuts from *Ginkgo biloba* have been used for hundreds of years to treat a wide variety of disorders, such as asthma, vertigo, tinnitus, as well as general circulatory problems (Cybulska-Heinrich et al., 2012). *Camellia sinensis* is a plant from which green tea can be produced. This beverage has a long traditional use as social drink but also as medicine in the treatment and prevention of disorders, dysfunctions, or diseases in humans and other animals (Batista et al., 2009; Moore et al., 2009). *Aesculus hippocastanum* (horse chestnut) is native to the countries of the Balkan Peninsula, but it is cultivated worldwide for its beauty. Historically, seed extracts from this plant have been used as a treatment for many ailments (Anonymous, 2009). *Crataegus monogyna* (hawthorn) is known as a traditional medicinal plant in many countries, growing in shrub communities and decidious thin forests (Öztürk and Tunçel, 2011). *Vitis vinifera* (grapevine) is an indigenous plant from southern and Western Asia, but it is cultivated today in all temperature regions of the world (Nassiri-Asl and Hosseinzadeh, 2009). Finally, *Allium cepa* (onion) is one of the most important vegetables worldwide and is extensively cultivated. It is an herbaceous bulbous plant that has a long tradition of being beneficial against inflammation, general cardiovascular diseases, and cancer (Slimestad et al., 2007).

Regarding knowledge on the chemical composition of plants, *Camellia sinensis* (green tea) is the plant with the highest number of chemical structures identified (710), followed by *Zea mays* (677) and *Panax ginseng* (601). Other chemically well characterized plants are *Citrus sinensis* (orange tree), *Apium graveolens* (celery), and *Daucus carota* (carrot), with 589, 533, and 507 known molecules, respectively. In contrast, many plants in the database have only one or very few endogenous metabolites identified, such as *Rhamnus alaternus* (Mediterranean blackthorn), *Lonicera etrusca* (honeysuckle), or *Hernaria glabra* (herniaria).

A detailed analysis of all the links between plants, metabolites, targets, and diseases in our integrated database (**Figure 1**) identified a total of 31,808 mechanistic hypotheses. At this stage, a mechanistic hypothesis is generated if a given plant known to have some therapeutic use contains at least one endogenous metabolite that is either known or predicted to interact with a human protein associated with its original therapeutic use. It ought to be stressed here that the concentration of any plant metabolite in a herbal preparation is very low and that, by any means, the results presented below imply that the metabolite assigned to the mechanistic hypothesis is the sole responsible of the therapeutic action of the plant but it will somehow contribute it. In this respect, a total of 893 mechanistic hypotheses for its different therapeutic uses could be generated for *Glycine max* (soybean). Among the plants with the highest number of mechanistic hypotheses generated, we found *Ginkgo biloba* (793), *Camellia sinensis* (781), *Citrus limon* (578), and *Vitis vinifera* (563). Out of the total number of 31,808 mechanistic hypotheses generated, 14,308 involved known interactions between endogenous metabolites and protein targets, whereas the remaining 17,500 hypotheses emerged from predicted interactions (see *Materials and Methods*).

### Retrospective Validation

**Figure 3:**
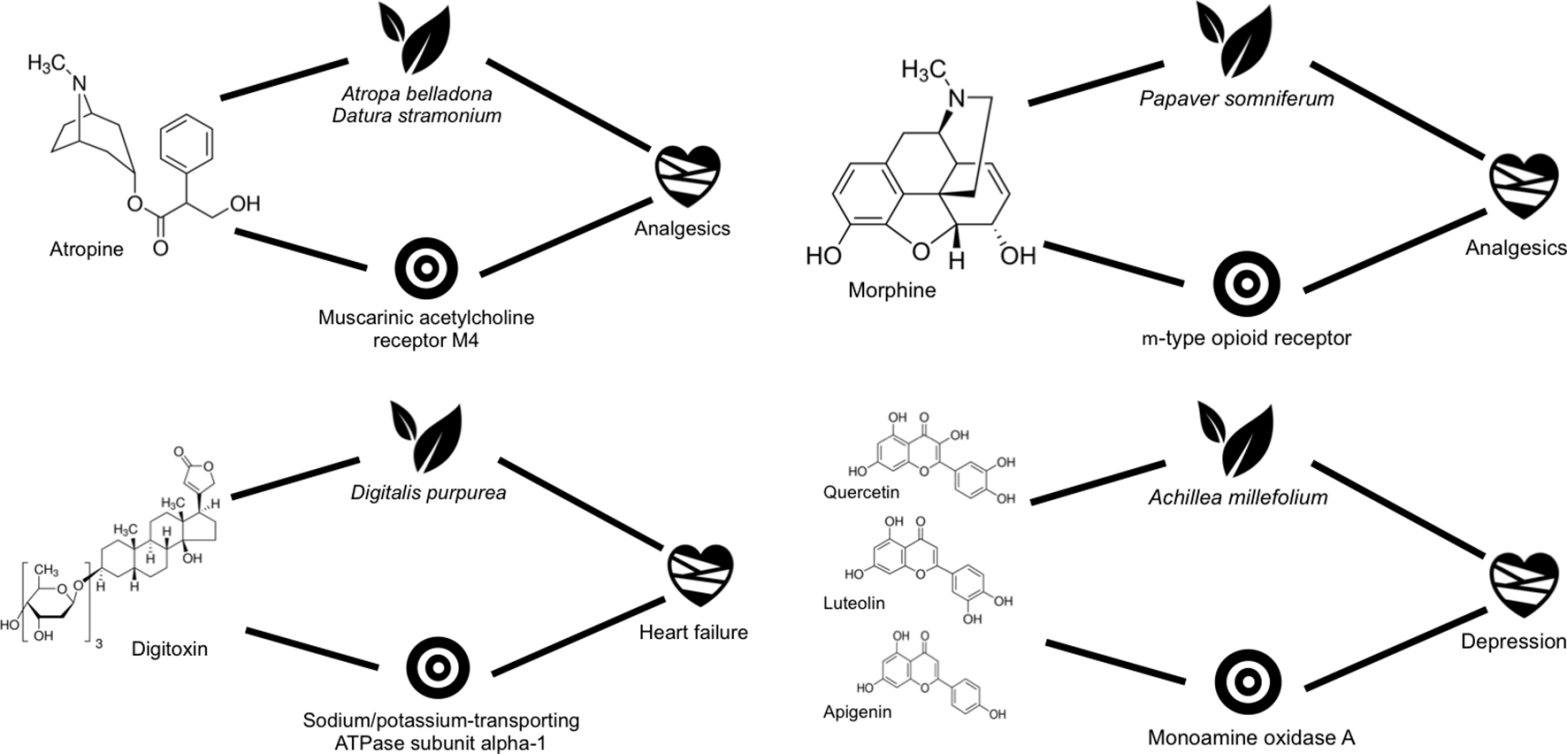
Scheme showing some of the closed circles confirmed retrospectively for some of the plants being used in cardiovascular diseases.

Among the molecules involved in the mechanistic hypotheses generated with known metabolite–protein interactions, we identified some well-known single active principles, such as atropine, morphine, and digitoxin, as well as a mixture of active principles, such as the one composed of quercetin, luteolin, and apigenin (**Figure 3**).

Atropine is found mainly in *Atropa belladonna* and *Datura stramonium* (Kurzbaum et al., 2001; Caksen et al., 2003), both used commonly for their analgesic action (Duttaroy et al., 2002; Overington et al., 2006). This molecule is known to be active against the muscarinic acetylcholine receptor M4, a therapeutic target associated with some analgesics. Therefore, we have all links described in **Figure 1** confirmed and thus forming a mechanistic hypothesis for the analgesic action of these plants (Soni et al., 2012; Owais et al., 2014).

Another widely recognized molecule for its analgesic activity is morphine. It is found in *Papaver somniferum* (opium poppy), and it was the first active alkaloid extracted from this plant (Jurna, 2003). Opium has been used in traditional medicinal as sedative and analgesic (Calixto et al., 2001). According to all links established in our database, morphine would be directly identified as a candidate to contribute to the analgesic action of opium through its interaction with µ-type opioid receptor (Choi et al., 2006; Yamada et al., 2006), a receptor well known for its association with analgesia (Inturrisi, 2002).

Digitoxin is a glycoside with known activity against the sodium/potassium-transporting ATPase subunit α-1, a protein associated with heart failure (Müller-Ehmsen et al., 2002; Hauck et al., 2009). Digitoxin is found in *Digitalis purpurea* (Chen et al., 2001), a plant used in traditional medicine for treating precisely this particular disease. Digitoxin has not only been proven to interact with the sodium/potassium-transporting ATPase subunit α-1, but it has also been shown to be indeed effective in heart failure (Belz et al., 2001).

Finally, we selected an example in which three compounds, namely, quercetin, luteolin, and apigenin, all confirmed endogenous metabolites of *Achillea millefolium* (yarrow), a plant used traditionally for treating depression, are known to have biologically relevant affinities for monoamine oxidase A (Lemmens-Gruber et al., 2006; Han et al., 2007; Benetis et al., 2008; Bandaruk et al., 2014), which, in turn, is one of the target proteins for depression (Thase et al., 1995).

**Figure 4:**
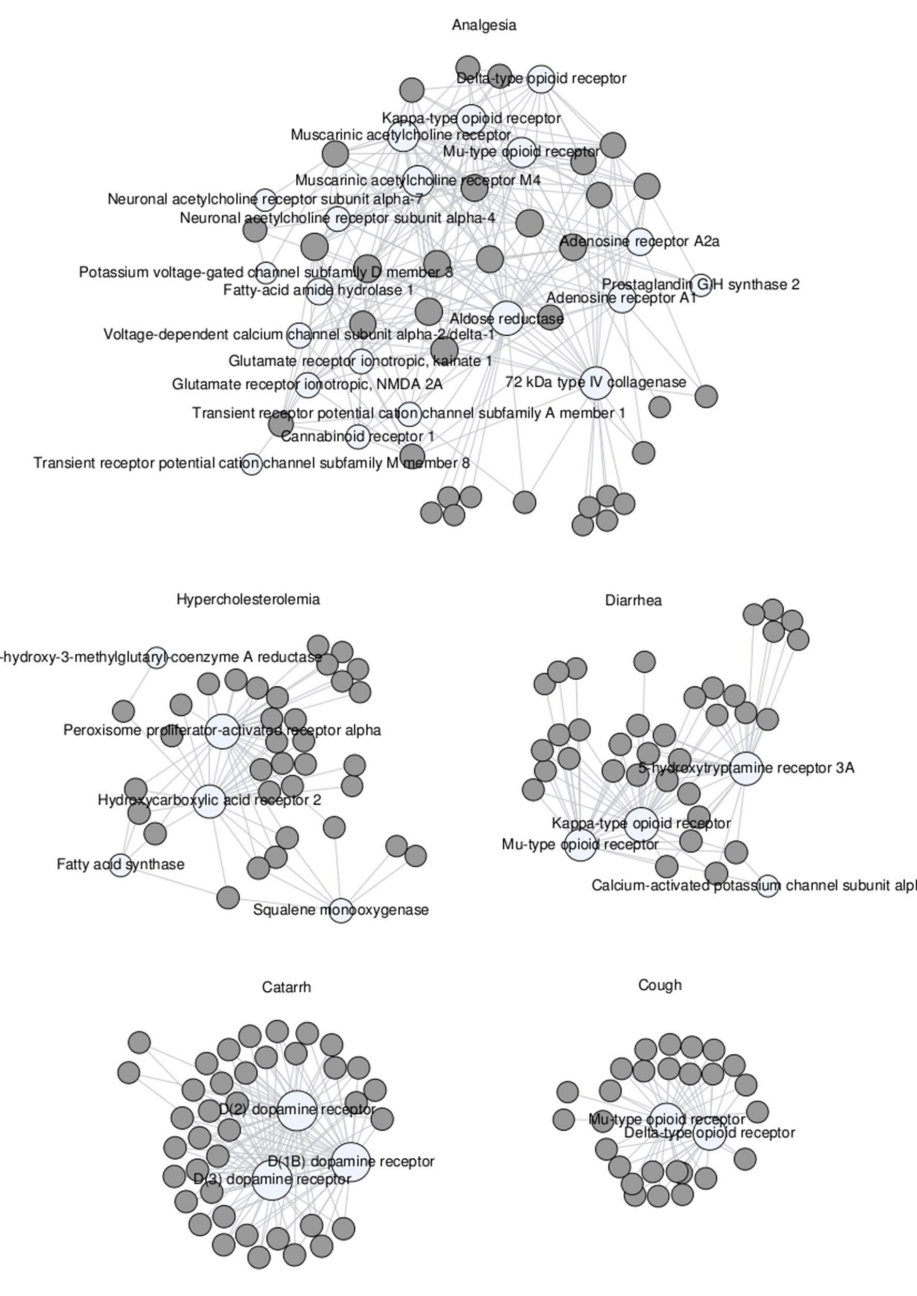
Network of remedial herbs (gray circles) linked to proteins (white circles) associated with various maladies, namely, analgesia, hypercholesterolemia, diarrhea, catarrh, and cough.

A more systematic analysis of all the mechanistic hypotheses that could be derived directly from known data and associations revealed that, among all disease categories, the circulatory, respiratory, and musculoskeletal systems collectively represented over 47% of all mechanistic hypotheses generated. The plant–protein networks derived for some specific diseases within these categories are shown in **Figure 4**.

In the analgesia network (**Figure 4**, top), one can observe that plants have multiple connections with a diverse range of proteins. This reflects the fact that some of the endogenous metabolites found in those plants have biologically relevant affinities for many of those proteins. Among them, aldose reductase (Young et al., 1983), muscarinic acetylcholine receptors (Duttaroy et al., 2002), and opioid receptors (Inturrisi, 2002) are the ones being most targeted by metabolites from these plants. Altogether, the formation of this complex network is indicative of a variety of plausible mechanisms of action relevant to analgesia, although one cannot exclude the possibility that the proteins involved in this analgesia network are closely related by cross-pharmacology, that is, they interact with similar ligands (Keiser et al., 2007; Briansó et al., 2011).

In contrast, the catarrh and cough networks (**Figure 4**, bottom) show that plants indicated for these therapeutic uses have endogenous metabolites targeting very much the same proteins. For catarrh, all plants contain some chemical entity that is active on the dopamine D1B, D2, and D3 receptors, all of them well known to be associated with respiratory diseases (Birrell et al., 2002). For cough, all plants contain at least one chemical entity with affinity for the µ and δ opioid receptors (Kotzer et al., 2000).

In between these two limit situations, the plant–protein networks obtained for hypercholesterolemia and diarrhea (**Figure 4**, middle) are consistent with different plants linked to these therapeutic uses acting through a small number of different mechanistic hypotheses. In this respect, most plants used for treating hypercholesterolemia seem to contain some chemical ingredient biologically active on the peroxisome proliferator-activated receptor α (56) and the hydroxycarboxylic receptor 2 (Karpe and Frayn, 2004). However, other plants may be exerting their therapeutic effect through interactions with some enzymes also associated with hypercholesterolemia, such as fatty acid synthase (Marseille-Tremblay et al., 2007) and squalene monooxygenase (Belter et al., 2011). Likewise, plants used for treating diarrhea contain chemical entities that have affinities for the µ and κ opioid receptors (Callahan, 2002), the 5-hydroxytryptamine 3A receptor channel (Sikander et al., 2009), and/or the calcium-activated potassium channel subunit α-1 (Deng et al., 2015). Even though many plants are targeting all of them, others seem to target only one or two.

It is important to highlight at this stage that the use of known data only is prone to the effects of completeness, and thus, several links may actually be missing in the networks discussed. In fact, looking at the distribution of the affinity values for those known interactions, we observe that in many cases these interacting chemicals are found also in plants that are not used for treating the disease associated with the interacting protein. Some of the reasons why these plants have not been used for these illnesses could be, for example, that the compound concentration is not enough in the plant or the plant really has this therapeutic action but it is simply not used for it. The same plant may have different uses in different parts of the world. Last but not least, it could well be that the action of some of these compounds requires the presence of some bioenhancer (Dudhatra et al., 2012), the therapeutic action being ultimately the result of multiple compounds acting synergistically. Overall, from the initial number of 372 plants associated with at least one therapeutic use, only 193 contain known data for all necessary links to derive a mechanistic hypothesis. Accordingly, the following section illustrates the use of high-confidence predictions as a means to enlarge the coverage of plants for which mechanistic hypotheses can be derived.

### Prospective Evaluation

**Figure 5:**
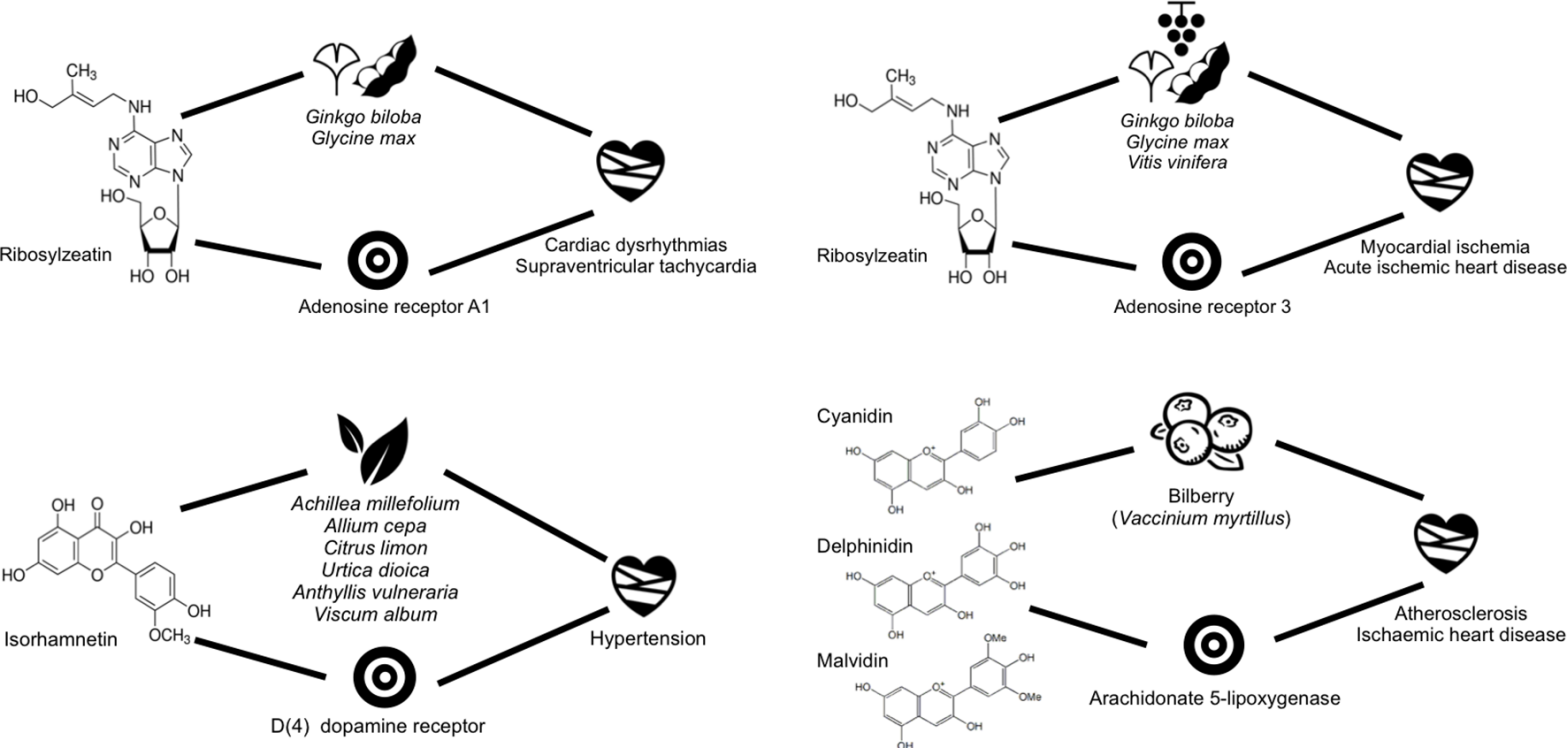
Scheme showing some of the closed circles confirmed prospectively for some of the plants being used in cardiovascular diseases.

Before embarking into the analysis of some of the mechanistic hypotheses emerging from predicted interactions, we validated the expected accuracy of those predictions for which known data was available. Overall, a good correlation was found between known and predicted affinity values for the same molecule–protein interactions. The median of the difference in affinities was 0.332, with 25% and 75% quartiles being at −0.1 and 0.7 with respect to the median, respectively, with a standard deviation of 0.794. Then, for those predicted interactions only, we focused on those providing a balance between potency of the predicted affinity and novelty of the prediction, as regarded by the similarity to the closest molecule for which the affinity for the same protein is known already. Among those, we prioritised the confirmation of the proposed mechanistic hypotheses for two single compounds, namely, rybosylzeatin and isorhamnetin, and one compound mixture, composed of cyanidin, delphinidin, and malvidin. The results are compiled in **Figure 5**.

Ribosylzeatin is an endogenous metabolite present in *Ginkgo biloba* (gingko), *Glycine max* (soybean), and *Vitis vinifera* (grapevine). This small molecule was predicted to have low micromolar affinity for the adenosine A1 and A3 receptors and *in vitro* testing performed subsequently confirmed 57% and 65% binding, respectively, at 10mM concentration. Accordingly, a mechanistic hypothesis could be derived suggesting that the interaction of ribosylzeatin with the adenosine A1 and A3 receptors may be contributing to the beneficial effects of those plants in the treatment of a number of cardiovascular diseases where these receptors are known to play a role, namely, cardiac dysrhythmias, supraventricular tachycardia, acute ischaemic heart disease, and mycocardial ischemia (Kiesman et al., 2009; Fishman et al., 2012). In this respect, soybean is known as an important source of proteins in diet, widely used as herbal medicine for the treatment of several cardiovascular diseases. Also, the cardioprotector properties of grapevine have been exploited in folk medicine since ancient times. In particular, the therapeutic action of grapevine against ventricular tachycardia was recently demonstrated in rats (Zhao et al., 2010). However, in this study, the authors used a proanthocyanidin grape seed extract. On the basis of the hypothesis generated here, we would suggest that ribosylzeatin is one of the active ingredients in grapevine participating in this therapeutic effect in synergy with other proanthocyanidins.

Isorhamnetin is a phytochemical present in multiple plants used for the treatment of hypertension. A low micromolar affinity between isorhamnetin and the dopamine D4 receptor was predicted. Upon *in vitro* testing, the experimental value obtained was only 28% binding. Despite this rather low affinity value, we could suggest that this compound may well be contributing to some extent to the effect on hypertension of the plants in which it is present. In fact, a similar chemical present in most of those plants listed and suggested to be partly responsible for their therapeutic action on hypertension is quercetin, reported to have also a low experimental affinity value of 5 µM against the same dopamine D4 receptor.

Finally, very much along the same lines reported above for the hypotheses generated from known metabolite–protein interactions, we were also keen on having a prospective mixture example. Accordingly, we predicted activity of cyanidin, delphinidin, and malvidin, all present in *Vaccinium myrtillus* (bilberry), on the arachidonate 5-lipoxygenase (ALOX5). *In vitro* testing of its mixture confirmed a 41% inhibition. This result provides a mechanistic hypothesis for the therapeutic use of bilberry for atherosclerosis and ischemic heart disease. It has been already suggested that quercetin is partially responsible for the therapeutic action of this plant due to its affinity for the ALOX5. We could add now delphinidin, cyanidin, and malvidin to the list of potential chemical effectors of this plant. Indeed, bilberry fruit contains high concentration of several anthocyanidins (Cassinese et al., 2007; Chu et al., 2011). In fact, other anthocyanidins present in bilberry, such as peonidin and petunidin, were also predicted to be active against this protein. So all these compounds may actually contribute synergistically to the therapeutic effect attributed to bilberry for the treatment of atherosclerosis and ischemic heart disease.

## Conclusions

An effort to integrate data linking metabolites, plants, diseases, and proteins has been shown to be useful to generate mechanistic hypotheses for some of the therapeutic uses of remedial herbs. In this respect, the use of predicted interactions largely increases our ability to generate mechanistic hypotheses for plants for which known data is scarce. This notwithstanding, one ought to admit that many computationally derived hypotheses may either be false positives or not truly contribute to the therapeutic effect exhibited by the medicinal plant. Unfortunately, it is impossible to pursue experimental confirmation of all hypotheses generated and offer general statistics of this limitation. Nonetheless, the examples presented offer clear potential for the use of this type of systems approaches to contribute to finding a scientific rationale for traditional medicines. There is much more to learn about nature and its use for therapeutic purposes, and more research in this direction is certainly necessary.

## Materials and Methods

**Linking plants to diseases.** A very first version of the database was created containing the therapeutic use of plants in traditional Catalan medicine (Gausachs, 2008). Plants were stored using their scientific name in Latin, whereas therapeutic uses were mapped to their corresponding disease identifier in ICD-10 (International Classification of Diseases Version 10). This initial database was complemented with additional therapeutic uses found for those plants in other public sources (Raja et al., 1997; Rigat et al., 2007; James et al., 2013). Data from the different sources was integrated using Latin names for plants and ICD-10 identifiers for diseases from 18 categories. In total, 372 medicinal plants associated with 187 therapeutic uses were collected at this stage (**Supplementary Material**).

**Linking plants to metabolites.** The endogenous metabolites identified at present for every single plant in the database were extracted from three different sources, namely, Dr Duke Phytochemical and Ethnobotanical Database (U.S. Department of Agriculture, Agricultural Research Service., 1992-2016), the KNApSAcK database (Nakamura et al., 2014), and Gausachs’ work on Catalan remedial herbs (Gausachs, 2008). Among those, only KNApSacK contains chemical structures linked to chemical names. Structures for the chemical names available only in the other two sources were extracted from PubChem (Bolton et al., 2008). The final set of chemical structures from all sources was unified and stored using InChI Keys. In the end, a total of 7,443 unique chemical structures present in 322 of those 372 medicinal plants could be gathered and added to the database (**Supplementary Material**).

**Linking proteins to diseases.** Next, a list of both known and explored human proteins associated with diseases was extracted from the Therapeutic Target Database (Zhu et al., 2012). These data was complemented with curated protein−disease links, with focus on cardiovascular diseases, available in the literature (Cases and Mestres, 2009). The final list of proteins was stored and unified using their UniProt identifiers (The UniProt Consortium, 2015). A final number of 724 unique proteins known to be relevant for 166 out of the initial 187 therapeutic uses were ultimately entered into the database (**Supplementary Material**).

**Linking metabolites to proteins.** Finally, affinity data (pK_i_, pK_d_, pIC_50_, pEC_50_) between chemical structures and proteins was extracted from various public sources (Roth et al., 2004; Bolton et al., 2008; Gaulton et al., 2012; Gilson et al., 2016; Southan et al., 2016). Up to 3,171 known interactions between 705 phytoconstituents and 228 proteins were identified and collected into the database at this stage. In addition, since affinity data are well recognised to be suffering from completeness issues (Mestres et al., 2008; Cases and Mestres, 2009), known interactions between molecules and proteins were complemented with high-confidence predictions obtained using ligand-based computational models implemented in the CT-link software (Vidal et al., 2011; Garcia-Serna et al., 2015). Accordingly, 16,897 additional interactions predicted between 2,796 molecules, present in 297 plants, and 313 proteins were generated. However, the vast majority of the interactions predicted were assigned relatively low confidence scores (CScore). If only high confidence predictions (CScore ≥ 0.7) are considered, a total of 1,555 predicted interactions remain. In the end, affinity data for a total of 4,726 interactions between 1,157 endogenous metabolites and 320 therapeutically relevant proteins were assembled and stored in the database (**Supplementary Material**).

**Predicting metabolite–protein interactions.** All metabolite two-dimensional structures were processed with the CT-link software to obtain predicted affinities for a list of over 2,000 protein targets (Vidal et al., 2011; Garcia-Serna et al., 2015). Predictions are based on six independent ligand-based approaches that include similarity-based methods, pharmacophore clusters, quantitative structure–activity relationships, machine learning techniques, and cross-pharmacology relationships (Vidal et al., 2011). Ligand-based target models were constructed from small molecules for which *in vitro* affinity data was publicly available (Bolton et al., 2008; Gaulton et al., 2012). Each prediction is assigned a confidence score (CScore) that depends on the number and type of methods as well as on the affinity range of the prediction (see **Supplementary Material**). In the last decade, predictions from CT-link have been validated both retrospectively (Vidal and Mestres, 2010; Flachner et al., 2012; Spitzmüller and Mestres, 2013) and prospectively (Areias et al., 2010; Mestres et al., 2011; Antolin et al., 2012; Montolio et al., 2012; Antolin and Mestres, 2015; van Voorhuis et al., 2016; Ellis et al., 2019) in a wide range of applications and therapeutic areas.

**Experimental *in vitro* assays.** For the prospective validation, two molecules and one herbal extract were selected for testing with *in vitro* assays at Cerep (CEREP Inc). Ribosylzeatin was tested in binding assays to confirm the predicted interactions with adenosine A1 and A3 receptors. Cellular assays were used to confirm the predicted interactions between isorhamnetin and the dopamine D4 receptor, as well as between a compound mixture (containing cyanidin, delphinidin, and malvidin) and 5-lipoxygenase.

For the binding assay, ribosylzeatin was tested twice at a test concentration of 10 µM. The reference agonist ligands used to calculate the compound activity were CPA for the adenosine A1 receptor and IB-MECA for the adenosine A3 receptor, which have IC_50_ values of 0.75 nM and 0.31 nM, respectively. The adenosine A1 receptor assay was performed in the presence of 1 nM of 3H.CCPA. After 60 min of incubation with shaking, bound radioactivity was separated from free by vacuum filtration and determined by scintillation counting. A similar procedure was followed for the adenosine A3 receptor assay. In this case, it was performed in the presence of 0.15 nM of 125I.AB-MECA. It was incubated with shaking during 120 min. After that, bound radioactivity was filtered and measured with scintillation counting. For these binding assays the results are expressed as a percent of measured specific binding relative to control specific binding.

For the dopamine D4 receptor assay, isorhamnetin was tested at a concentration of 10 µM. The reference agonist ligand was dopamine, with an EC_50_ value of 28 nM. D4.4 was incubated for 10 min at 37ºC and, after that, cAMP was detected and measured with HTRF. Results are expressed as a percentage of measured response relative to control response.

Finally, for the testing of the compound mixture of cyanidin, delphinidin, and malvidin in the 5-lipoxygenase enzyme assay, the reference compound used was NDGA, which has an IC_50_ of 910 nM. 5-Lipoxygenase was incubated 20 min with shaking and 25 µM arachidonic acid as substrate. Thereafter, rhodamine 123 was measured using fluorimetry. Results are expressed as a percentage of measured specific binding relative to control specific binding.

Compounds showing an inhibition or stimulation higher than 50% were considered to be active for the proteins tested, whereas interactions showing activity values between 25% and 50% were considered to be indicative of at least weak to moderate effects.

## Data Availability Statement

All datasets generated for this study are included in the manuscript/**Supplementary Files**.

## Author Contributions

JM conceived and designed the study. JO acquired the data for the analysis. JO and JM analyzed and interpreted the data, discussed the results, and wrote the manuscript.

## Funding

This research was supported by the Spanish Ministerio de Ciencia Innovación y Universidades (project SAF2017-83614-R).

## Conflict of Interest

The authors declare that the research was conducted in the absence of any commercial or financial relationships that could be construed as a potential conflict of interest.

## Supplementary Material

Supplementary Table 1 Collected data connecting diseases and plants, plants and metabolites, metabolites and proteins, and proteins and diseases. Also provided are the different subsets used to construct the plant-disease and plant-protein networks of Figures 2 and 4, respectively.
